# The impact of pre-processing techniques on deep learning breast image segmentation

**DOI:** 10.1038/s41598-025-30724-9

**Published:** 2025-12-16

**Authors:** Jéssica Catarino, Nuno Cruz Garcia, Sara Silva, João Santinha

**Affiliations:** 1 LASIGE, Faculdade de Ciências, Universidade de Lisboa, 1749-016 Lisbon, Portugal; 2 Digital Surgery Lab, Breast Cancer Research Program, Champalimaud Foundation, 1400-038 Lisbon, Portugal; 3 Faculty of Medicine, University of Lisbon, 1649-028 Lisbon, Portugal

**Keywords:** Breast cancer, Computational models, Image processing, Machine learning, Medical imaging

## Abstract

Breast cancer is one of the most common forms of cancer worldwide, making breast imaging a critical area for developing and evaluating Deep Learning methods. In this study, we investigate how different pre-processing techniques influence model performance in breast image segmentation. Pre-processing is a crucial step in the Deep Learning pipeline that directly impacts model performance, yet studies on its role in medical imaging remain limited. We assess the influence of different pre-processing techniques on a U-Net segmentation model applied to two breast public imaging datasets: CBIS-DDSM and Duke-Breast-Cancer-MRI. We systematically explored commonly used methods, including pixel intensity normalization, spacing harmonization, resizing/padding, and orientation standardization. Two processing pipelines were developed: Domain Non-Specific, integrating standard practices from natural and medical image analysis, and Domain Specific, which preserves anatomical information through careful handling of breast imaging metadata. A detailed comparative analysis of each pre-processing technique was conducted to evaluate its impact on model performance. Despite challenges and limitations associated with dataset size and scope, our findings identify pre-processing strategies tailored for breast imaging that can improve segmentation accuracy and analysis. This study represents an initial step in evaluating pre-processing for medical image analysis, providing a foundation for future work. Our results highlight significant differences in a 3-way ANOVA F-test ($\alpha < 0.05$) for U-Net segmentation outcomes, attributed to different pixel intensity normalization approaches, offering valuable insights for future research.

## Introduction

After the success of Convolutional Neural Networks (CNNs) in the 2012 ImageNet challenge^1^, the use of this family of Deep Learning (DL) architectures was naturally adopted in radiology, where medical images provide diverse insights into patient anatomy. For example, when clinicians suspect a cancer lesion, they may use advanced imaging techniques to obtain a more precise diagnosis^2,3^.

The images in ImageNet are non-radiological and consist of objects at different scales, orientations, and acquisition conditions. Consequently, DL model training typically involves resizing images to a uniform size for computational efficiency and harmonizing pixel intensities to ensure model convergence. Pre-processing transformations for ImageNet, which also serve for data augmentation, do not need to preserve scale or orientation and can independently alter color, intensity, and contrast. Although these transformations do not maintain the scale and intensity information, critical in medical images, they have been adopted in medical imaging DL pipelines. However, and specifying this context to breast imaging, different modalities have distinct properties (e.g., 2D mammography vs. 3D tomosynthesis vs. 3D Magnetic Resonance Imaging (MRI), quantitative vs. non-quantitative image intensities) that influence pre-processing requirements.

The evolution of breast imaging modalities over the years has enabled visualization of anatomy in different dimensions, including two-dimensional (2D) imaging such as digital mammography, three-dimensional (3D) imaging like breast tomosynthesis or volumetric MRI, and four-dimensional imaging (4D; 3D imaging over time – e.g., Dynamic Contrast-Enhanced MRI (DCE-MRI) – or 3D with different acquisition parameters – e.g., diffusion-weighted imaging (DWI))^3^. In breast imaging, two-dimensional segmentation tasks, such as those based on mammography, involve planar images with limited spatial context, whereas three-dimensional segmentation tasks, including breast tomosynthesis and volumetric MRI, require handling volumetric data with precise spatial relationships, which influences pre-processing strategies, model design, and segmentation performance.

Breast images can also be categorized by the meaning of their pixel values. Quantitative imaging employs standardized measurement units, facilitating uniform analysis. For example, MRI Apparent Diffusion Coefficient (ADC) maps in DWI provide quantitative measures of water diffusion within breast tissue, which can help differentiate malignant from benign lesions^2,4^. Similarly, DCE-MRI provides dynamic information on contrast uptake, which is valuable for tumor characterization and treatment monitoring. Non-quantitative modalities, such as standard mammography, rely on intensity contrasts between fatty and fibroglandular tissue to detect lesions.

Breast images can also be categorized by the meaning of their pixel values. Quantitative imaging employs standardized measurement units, facilitating uniform analysis. For example, MRI allows acquisition of quantitative images such as ADC maps in DWI and relaxation time maps (T1, T2, T2*). ADC maps provide quantitative measures of water diffusion within breast tissue, which can help differentiate malignant from benign lesions^2,4^. Similarly, DCE-MRI provides dynamic information on contrast uptake, which is valuable for tumor characterization and treatment monitoring. Non-quantitative modalities, such as standard mammography, rely on differences between fat and fibroglandular tissue to detect lesions.

In contrast, non-quantitative images enable visual assessment of tissue contrasts without providing specific numerical values for each structure. This approach relies on interpreting variations in pixel intensities to identify different tissues, as in mammography, standard MRI sequences, and breast ultrasound.

Furthermore, like other medical images, breast imaging data are typically stored in specialized file formats that include metadata, such as image geometry and, in some cases, patient, hospital, physician, and acquisition details. The Digital Imaging and Communications in Medicine (DICOM) format contains all of this information and is the standard in clinical settings^5^. In contrast, research-oriented formats such as Neuroimaging Informatics Technology Initiative (NIfTI) and Nearly Raw Raster Data (NRRD), among others, generally include only image geometry information^6^. Despite the availability of these specialized file formats, some datasets still have medical images converted to other formats like Portable Network Graphics (PNG) or Joint Photographic Experts Group (JPEG), resulting in the loss of necessary metadata associated with the image. This can create challenges for researchers and artificial intelligence developers who require complete and accurate information about the image for their pre-processing and analysis^5^.

Among the image geometry information that may require different pre-processing steps and may affect the machine learning model performance are:

- **Matrix size** refers to the number of pixels (2D) or voxels (3D) in each dimension, which often varies within datasets due to different sources or radiologist adjustments^7^. Such variations complicate data preparation for analysis, particularly with DL methods, as they require uniform image sizes. Choosing a consistent matrix size for all images is crucial, considering the model’s input needs and available computational resources. High-resolution images demand more memory and power, posing challenges requiring image resizing or cropping for dataset consistency. This process must balance preserving image quality for accurate diagnosis and fulfilling computational model requirements.
- **Pixel/voxel spacing** indicates the size of each pixel or voxel in the image’s spatial dimensions, essential for understanding image grid dimensions^8^. Given that medical images vary in metadata, including pixel size, due to different production methods, normalizing and standardizing this data becomes crucial for tasks like segmentation or feature extraction^9^. It is important to consider pixel/voxel spacing variations, as inconsistencies can lead to misleading comparisons, inaccurate anatomical structures, or differences in feature measurements between images from different sources. Considerable variability may influence the model’s ability to segment anatomical structures in the images.
- **Orientation** in medical imaging refers to how elements are arranged, both their physical layout and their arrangement relative to each other, focusing on the axial plane for radiological analysis. Medical images often differ from clinicians’ preferred viewing setup due to traditional conventions^6^. These images, stored in 2D slices, combine to represent a 3D structure, each slice containing metadata about its orientation relative to real-world anatomy. Ensuring consistent orientation is crucial as discrepancies can lead to errors in image-segmentation pairing, affecting the training and reliability of DL models^6^.

Therefore, using medical image domain knowledge about these image properties can be used to ensure that pre-processing prepares images for DL models without distorting the observed distribution of anatomies. Additionally, these pre-processing pipelines may be important to avoid adding confounders and creating undesirable model behaviors (e.g., biases, overfitting, underfitting). Recently, new frameworks have been developed that include internal modules to build a ”data fingerprint” and automatically apply appropriate transformations based on the characteristics of the dataset, like nnU-Net^10^ and Auto3DSeg^11^.

These frameworks are valued for their simplicity, as they embed domain-specific pre-processing steps and automate parameter adjustment. However, when developing or testing architectures outside these frameworks, pre-processing must often be defined manually, which can lead to inconsistencies. This highlights the continued importance of understanding and optimizing pre-processing in DL training pipelines^12^.

Several studies have assessed the influence of different individual pre-processing techniques, evaluating their impact on DL models, such as Sabottke et al.^13^, where image resolution was investigated, and Jia et al.^14^, where different image normalization algorithms were used and their impact on the performance of the DL classification was assessed. In most studies in the field of DL, other pre-processing methods were used, but mainly for data augmentation purposes^15^.

In this study, we perform an extensive comparison of the impact of several pre-processing methods to better understand the impact and importance on DL model performance. We investigate whether a set of more adequate methods exists for breast imaging DL segmentation, taking into account the unique characteristics of these images, particularly in light of the lack of established guidelines for such pre-processing in the literature.

The choice of breast imaging and segmentation tasks was motivated by the societal impact of breast cancer, one of the most common cancers worldwide, and by the central role of multimodality imaging in screening, diagnosis, and treatment assessment. In this study, we focused on mammography and breast MRI, as mammography is widely used for 2D screening, while MRI provides 3D volumetric data for detailed tissue characterization. Additionally, the availability of curated breast imaging datasets with detailed annotations of tissues and lesions enabled us to systematically evaluate the impact of pre-processing across different modalities and regions of interest with distinct features.

Besides the fact that there are many more pre-processing approaches that can be used to transform the input data, we developed two distinct pre-processing pipelines using some of the most frequent and commonly used transformations: one, called *Domain Non-Specific* (DNS), that applies conventional transformations without preserving anatomical consistency (e.g., orientation, pixel/voxel spacing and intensity, and image size) and another, called Domain Specific (DS), that explicitly maintains these spatial and intensity characteristics, as show on Table 1. Within each pipeline, we systematically evaluated different combinations of the selected pre-processing techniques and assessed their impact on model performance across two breast imaging modalities.

**Table 1:** Overview of key distinctions in pre-processing strategies between domain non-specific images (e.g., natural images) and domain-specific images (e.g., medical images).

Parameters	Domain non-specific	Domain specific
Orientation	No standardization	Same orientation
Pixel Spacing	No standardization	Median value of each axes
Pixel Intensity	Minimum–maximum	Percentiles, Z-Score, Histogram Normalization, and others
Image Size	Resize to square shapes	Resize maintaining aspect ratio OR Padding and resizing

The table highlights differences in handling image size, pixel intensity, pixel spacing, and orientation.

## Materials and methods

In this section, we start by presenting the breast datasets used. Then, we present the pre-processing methods that are part of the pipelines and detail the two data processing pipeline types: (anatomy/physiology) non-preserving and preserving. Lastly, we describe our detailed comparison of the pre-processing steps and focus on the model architecture and evaluation.

### Breast datasets

For this work, we have decided to explore two different breast image modalities: mammography (CBIS-DDSM Dataset^16^) and MRI (Duke-Breast-Cancer-MRI Dataset^17^), which we will elaborate on in detail.

**Curated Breast Imaging Subset of Digital Database for Screening Mammography Dataset** (CBIS-DDSM) is a database that contains about 2,620 scanned film mammography cases. CBIS-DDSM includes mammographic (with two images per view: Craniocaudal (CC) and Mediolateral Oblique (MLO)) images with annotations for mass and calcification cases, BI-RADS assessments, pathology-confirmed diagnoses, and ROI segmentation masks. The images are provided as full-field digital images in DICOM format, with variable resolutions, ranging from 2000 $\times$ 5000 to 4800 $\times$ 6400 pixels, ensuring high detail for lesion analysis^18^.

**Duke-Breast-Cancer-MRI Dataset** features a comprehensive collection of 922 DICOM breast MRI sequences from Duke University Medical Center’s patients with biopsy-confirmed invasive cancer. Patients were positioned face down for their scans, utilizing either a 1.5 Tesla or 3.0 Tesla machine from GE Healthcare or Siemens. The technique captured horizontal cross-section images, with the voxel dimensions varying between 320 $\times$ 320 $\times$ 144 and 512 $\times$ 512 $\times$ 200 pixels. This dataset is distinguished by its inclusion of 3D bounding boxes around lesions and detailed segmentations of the breast, fibroglandular tissue, and vascular structures. These features serve as a valuable tool for researchers in cancer detection, tissue segmentation, and analysis, facilitating accurate studies of tumor location, size, and the surrounding anatomy^17^. For this study, we used 100 annotated patients from the Duke Breast Cancer MRI dataset, specifically selecting subjects with available pre-contrast fat-saturated T1-weighted images and manual annotations for breast tissue, fibroglandular tissue (FGT), and blood vessels. The 100 patients were randomly selected from the full dataset by Zhang et al.^19^, and all annotations were created using 3D Slicer and reviewed by board-certified breast radiologists to ensure accuracy and consistency. The patients selected are shown in Supplementary Table S1.

### Pre-processing methods

DL models require standardized inputs, which are obtained by pre-processing the images before they are given to the network. This pre-processing involves intensity normalization, resizing, and other harmonization techniques that ensure optimal input quality, which can impact model performance, such as segmentation accuracy, training time, or generalization ability.

A set of primary pre-processing techniques that are often applied to medical images is presented here:

- **Harmonization of pixel spacing and intensity**, as these can vary significantly, arising from the diversity of settings and calibrations of medical imaging equipment used across different institutions, but also due to different anatomic characteristics of each patient. Pixel spacing denotes the physical distance between the center of each pixel in an image, which affects the scale of the observed anatomy and is often harmonized through resampling techniques^8^. On the other hand, pixel intensity represents the brightness levels within the image, which must be standardized to ensure consistent interpretation of the different tissues^8^. While for quantitative images, the harmonization of intensities between -1 and 1 or 0 and 1 may be needed to ensure faster convergence and training of the models, in non-quantitative images, selecting a proper intensity harmonization method is more difficult. In this scenario, several methods can be applied to standardize the pixel intensity values, such as minimum-maximum (Min-Max) and percentile-1–percentile-99 (P1-P99) methods^20^.
- **Padding, cropping, and resizing** are often used to ensure that all images in a dataset have the same dimensions to facilitate the application of deep learning algorithms. Padding involves adding a pixel border around the image to increase the size without altering or distorting the original content, preserving the aspect ratio. Contrarily, cropping reduces the image size by removing its outer parts. It can help focus on the essential parts of the image or eliminate unnecessary parts. Unlike the previous two, resizing changes the image dimensions to fit a specified size, which may involve stretching or compressing the image content.
- **Orientation** is often used for data augmentation purposes to generate additional data but with slight variations through changes in position or other kinds of transformation^21^. However, when it comes to medical imaging, it is essential to ensure accurate image orientation, preserving the integrity of diagnostic quality for different clinical procedures^22^. Current auto-encoders do not offer orientation-invariant features. As these methods are sensitive to changes in orientation, image rotations or flips can change their representation in the model’s embedding space, which may have some impact on computational tasks^23^.

For this study, dimension transformations, such as changes to pixel spacing and matrix size, and pixel intensity transformations, will be our focus. Despite the existence of other pre-processing methods^24^, the choice was made considering the grayscale nature of medical images and the transformations that can visually distort the pixel/voxel information of the patient.

### Anatomy/physiology non-preserving transformation pipelines: domain non-specific (DNS)

For the CBIS-DDSM and Duke-Breast-Cancer-MRI datasets, we applied common transformations typically used in natural imaging, including resizing and standard normalization techniques^21,25^. Table 2 presents an overview of the pipeline created. Specifically, for the CBIS-DDSM dataset, we preserved the original orientation of mammography images and respective ground truth segmentations, ensuring that the images of the left breast faced leftward and those of the right breast faced rightward. After this, we added the pre-processing generally used in computer vision: normalizing pixel intensity values and resizing the images to a square shape. For the Duke-Breast-Cancer-MRI dataset, we first recreated the 3D volumes from the DICOM files and saved the volumes in NIfTI without changing intensity.

Afterward, we standardized the number of slices in the 3D volumes by considering the 64 central slices required due to computational constraints. Subsequently, we performed the pixel intensity harmonization and resizing of the images, typically used in computer vision tasks.

**Table 2:** Domain non-specific (DNS) pre-processing pipeline, which does not preserve anatomical structure information.

Order	Parameter	2D mammography models	3D breast MRI models
1st	Orientation	No standardization	No standardization
2nd	Pixel Spacing	No standardization	No standardization
3rd	Pixel intensity	Minimum–maximum, percentiles, Z-Score, Histogram Normalization	Minimum-Maximum, Percentiles, Z-Score, Histogram Normalization
4th	Image Size	256 × 256, 512 × 512, 1024 × 1024 256 × 448, 512 × 896, 1024 × 1792	256 × 256 × 64, 512 × 512 × 64

The table shows the order of pre-processing steps and the corresponding methods evaluated for two datasets composed of: (a) 2D mammography and (b) 3D breast MRI. The steps include image resizing, pixel intensity normalization (Min-Max, P1-P99, Z-Score, HistNorm), pixel spacing, and orientation.

### Anatomy/physiology preserving transformation pipelines: domain specific (DS)

Based on the characteristics of breast images, their metadata, and the known importance of pixels/voxel values in these images, some existing pre-processing methods can distort anatomical information or even result in losing essential details. There is little investigation into how those distortions affect the model’s ability to segment lesions or other tissues. Each modality has specific parameters crucial for standardizing information while carefully avoiding damage to the pixels/voxels. In some studies, there is a growing interest in adapting the pre-processing steps to the medical imaging modalities, yet without empirically testing the effectiveness of such methods^26^. Furthermore, the work by Masoudi et al.^27^ confirmed that appropriate pre-processing of medical images and the correct sequencing of each method can enhance the performance of segmentation models. With this in mind, we propose a set ordered of pre-processing methods to standardize the images more effectively and reduce the likelihood of negatively affecting model training and final performance. A scheme of this pipeline is presented in Table 3.

For the CBIS-DDSM dataset, we started by using the same images used in the DNS pipeline described above, ensuring that all images have the same orientation, as in mammography, both breasts pointed in the same direction. Also, we made sure we had uniform pixel spacing, which was already the case. To account for variations in image sizes, we padded smaller images to match the largest ones in the dataset (3328 $\times$ 4084). We also normalized pixel intensity across the dataset to address the variations across scanner manufacturers and models. Finally, we resized the images to a smaller dimension to accommodate computational limitations often observed with such large images. This resizing was performed only after making all size adjustments, and to ensure we were not deforming the anatomical structures of patients.

For the Duke-Breast-Cancer-MRI dataset, we also started by using the same images as in the DNS pipeline. We standardized image orientation, ensuring models received images always in the same direction.

We harmonized voxel spacing across our dataset by resampling all images to the median spacing obtained from the training set, similar to Isensee et al.^28^. The voxel intensity normalization techniques followed the same approach as the one taken for the CBIS-DDSM dataset. Finally, we resized the images to mimic computational and model constraints and determine if a particular input size could be more beneficial for the model.

**Table 3:** Domain-specific (DS) pre-processing pipeline, designed to preserve anatomical structure.

Order	Parameter	2D mammography models	3D breast MRI models
1st	Orientation	All breasts pointed in the same direction	Right Anterior Superior (RAS)
2nd	Pixel Spacing	Already isotropic	Median value of each axes
3rd	Pixel Intensity	Minimum-Maximum, Percentiles, Z-Score, Histogram Normalization	Minimum-Maximum, Percentiles, Z-Score, Histogram Normalization
4th	Image Size	Padding and resizing to 256 × 256, 512 × 512, 1024 × 1024	256 × 256 × 64, 512 × 512 × 64

The table shows the order of application and corresponding methods for each step, including orientation standardization, pixel/voxel spacing harmonization, pixel intensity normalization (Min-Max, P1-P99, Z-Score, HistNorm), and resizing. For (a) 2D mammography, resizing was applied either with padding to the maximum original image size or directly without padding, while for (b) 3D breast MRI, volumetric resizing was performed.

### Experiments

To study the impact of pre-processing on the development of medical imaging segmentation models, we conducted two levels of assessments. First, we compared the two pipelines, DS and DNS, to evaluate how a pre-processing pipeline, designed to preserve or not preserve anatomical information, affects a DL segmentation model. Second, we assessed each pre-processing step, including image orientation, pixel spacing, intensity normalization, and resizing/padding, to determine their impact on the segmentation model.

For the pre-processing pipeline comparison, we started by assessing the impact of standardizing the orientation of images in each dataset. For the CBIS-DDSM dataset, we aimed to standardize the orientation so that all breast images faced the same direction. In the case of the Duke-Breast-Cancer-MRI dataset, we standardized all images to the RAS orientation (Right Anterior Superior). Next, we evaluated the effect of harmonizing the pixel/voxel spacing across images within each dataset. For the CBIS-DDSM dataset, adjusting the spacings was unnecessary, as all images within this dataset already shared the exact dimensions (1.0 mm $\times$ 1.0 mm). However, the Duke-Breast-Cancer-MRI dataset presented variable pixel spacings across its images. Therefore, we conducted a preliminary analysis of the training set’s voxel spacings, determining the median values for each axis (0.7031 mm $\times$ 0.7031 mm $\times$ 1.0000 mm). Following this, we explored different methods for normalizing pixel/voxel intensity, using different techniques available in MONAI (version 1.3.0)^20^: Min-Max, P1-P99, Z-Score, and Histogram normalization.

For images from the CBIS-DDSM dataset, given their rectangular shape, we applied padding to convert them into square shapes, followed by resize transformations (256 $\times$ 256, 512 $\times$ 512, and 1024 $\times$ 1024) to evaluate the impact of avoiding the images’ distortion (either stretching or squeezing). Additionally, we explored the effect of maintaining the images’ rectangular shape by merely resizing them to assess the impact of this processing approach (256 $\times$ 448, 512 $\times$ 896, and 1024 $\times$ 1792).

In the Duke-Breast-Cancer-MRI dataset, with a maximum width $\times$ height of 512 $\times$ 512, we experimented with resize transformations of 256 $\times$ 256 $\times$ 64 and 512 $\times$ 512 $\times$ 64 to determine their effectiveness.

### Model architecture and evaluation

We used a U-Net model from the MONAI framework^20^ with the same hyperparameters and configurations in all experiments. The model architecture and training parameters used are shown in Table 4. We trained the models using a 5-fold cross-validation approach, keeping the split consistent to ensure a standardized basis for comparing and evaluating our models’ performance across experiments. We divided the data into train/validation sets by patient identifier before any analysis, transformation, or hyperparameter selection to avoid data leakage. For the CBIS-DDSM dataset, we split the data from 1152 patients into 5-fold: four for training and one for validation. Each fold was used as the validation set, repeating the process five times. For the Duke-Breast-Cancer-MRI dataset, we used the 100 patients with segmentations publicly available, splitting them with the same process as the CBIS-DDSM dataset.

**Table 4:** Some model training parameters changed using the CBIS-DDSM and Duke-Breast-Cancer-MRI datasets, and the Reduce On Plateau parameters for the Learning Rate (LR) refinement.

Dataset	Initial LR	LR Factor	LR Patience	LR Threshold	Channels	Strides
CBIS-DDSM	0.0001	0.7	100	0.0001	(16, 32, 64, 128)	(2, 2, 2)
Duke-Breast-Cancer-MRI	0.001	0.7	100	0.0001	(16, 32, 64, 128)	(2, 2, 2)

All the models were trained with a batch size of 4 and for 700 epochs.

Our goal is to evaluate the influence of each pre-processing step on the segmentation models and determine whether one pipeline produces better results than the other. We employed an initial learning rate of 0.0001 and used a Reduce On Plateau learning rate scheduler, with the factor, patience, and threshold specified in Table 4. Specifically, we focused on lesion segmentation for the model using CBIS-DDSM images, while for the Duke-Breast-Cancer-MRI dataset, we aimed to segment both breast and fibroglandular tissues and vessels using the existing annotations in each dataset. To evaluate the models, we calculated the Dice Similarity Coefficient (DSC) and Hausdorff Distance (HD) metrics, along with their respective standard deviations (STD), using the Segmentation Metrics Package (version 1.2.7)^29^, prior to resizing the images back to their original pixel intensities and spacings.

We trained the models for 700 epochs, storing the best epoch model checkpoint of each fold and respective metrics.

For the CBIS-DDSM dataset, we obtained the mean validation DSC and HD of the 5-fold and respective STDs. For the Duke-Breast-Cancer-MRI dataset, we calculated the same metrics for all labels separately, the mean of the three labels (breast, fibroglandular tissue, and vessels), and the respective STD.

Finally, we conducted an ANOVA F-Test^30^, from the Pingouin library (version 0.5.5), specifically a 3-way/4-way ANOVA to assess whether there are statistically significant differences among the trained models, setting statistical significance to $\alpha < 0.05$. This analysis aimed to identify which pre-processing steps impact the models most. We performed pairwise comparisons to evaluate specific differences within each parameter using the T-test with the Holm-Bonferroni multiple comparisons correction method^30^.

## Results

**Fig. 1:**
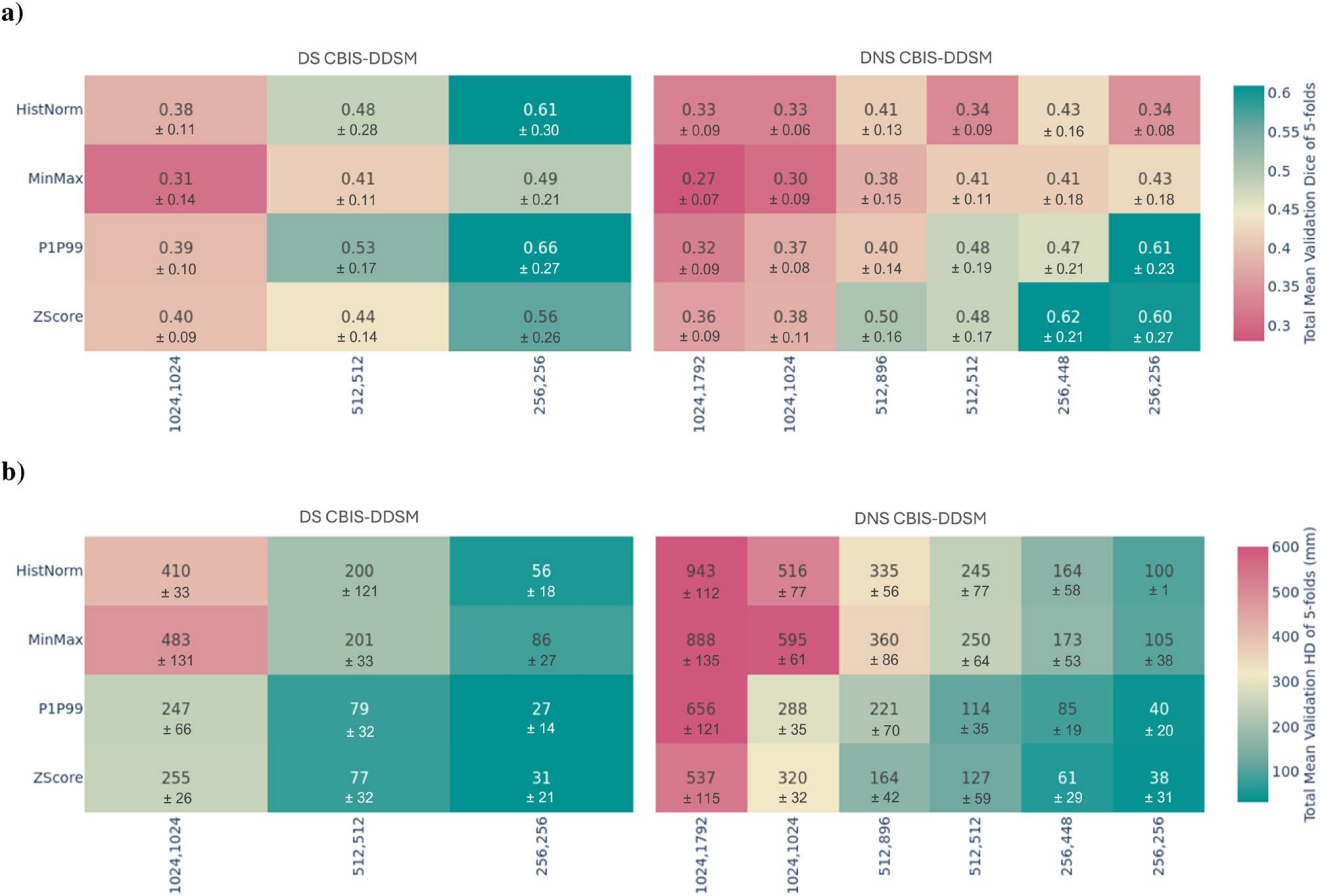
Results from the Domain-Specific (DS) and Domain Non-Specific (DNS) pipelines using the CBIS-DDSM dataset: (**a**) mean validation Dice Similarity Coefficient and (**b**) mean Hausdorff Distance across 5 folds for models with different resizing methods and pixel intensity normalization, shown with their respective standard deviations. DS and DNS models differ only in resizing and normalization parameters.

**Fig. 2:**
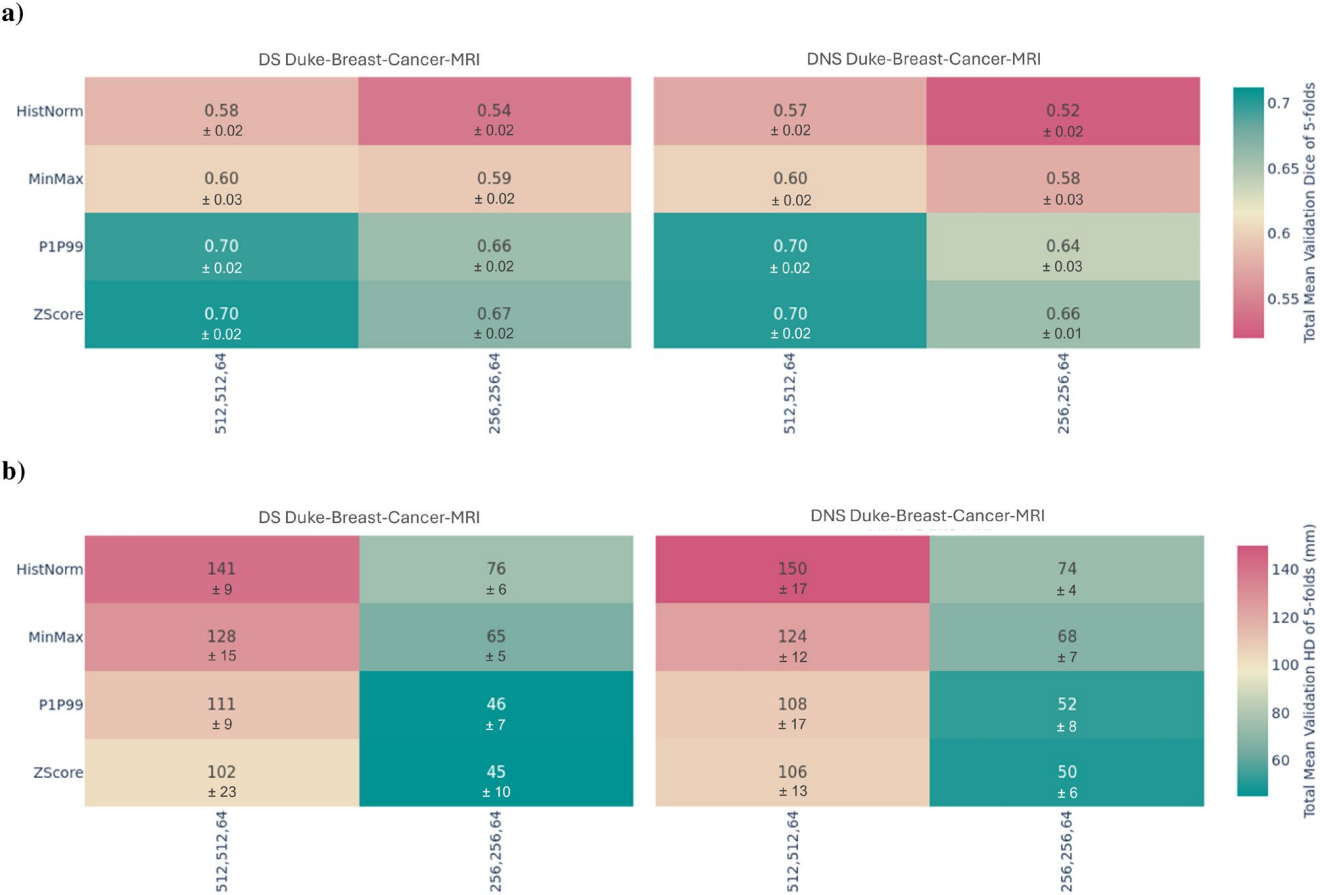
Results from the DS and DNS pipelines using the Duke-Breast-Cancer-MRI dataset: (**a**) mean validation Dice Similarity Coefficient and (**b**) mean Hausdorff Distance across 5 folds for three labels (breast, fibroglandular tissue, and vessels), shown with their respective standard deviations. The DS pipeline includes orientation and pixel spacing standardization during preprocessing, whereas the DNS pipeline does not.

To analyze the impact of pre-processing steps on model performance, we obtained heatmaps summarizing our findings. Figures 1 and 2 highlight the influence of pixel intensity normalization and resize steps for the CBIS-DDSM and Duke-Breast-Cancer-MRI datasets. These two pre-processing steps were selected due to the observed impact. The other pre-processing steps are described in the detailed comparison Tables 5, 6, and 7.

The first set of heatmaps, *a)*, in Figs.  1 and  2, illustrate each model’s final validation DSC, highlighting the influence of pixel/voxel intensity normalization and resizing, and the different methods tested at each step across the DS and DNS pipelines. For the CBIS-DDSM, the lesion DSC is presented, while for Duke-Breast-Cancer-MRI, we show the mean DSC of breast, fibroglandular tissue, and vessels. The second set of heatmaps, *b)*, presents the HD using the same configurations as in *a)*. These visualizations comprehensively overview how pre-processing methods affect segmentation performance.

### Pipelines

The heatmaps show differences between the DS and DNS models across both the CBIS-DDSM and Duke-Breast-Cancer-MRI datasets. These differences were confirmed through 3-way and 4-way ANOVA F-tests, which showed significant effects ($\alpha < 0.05$) for pixel intensity normalization, resize, and the combined preprocessing steps. Specifically, for the CBIS-DDSM dataset, resize had the largest effect, followed by pixel intensity normalization and pipeline, with significant two-way and three-way combinations among these factors, indicating that the joint application of pre-processing steps influences model performance beyond individual effects. This pattern was observed in both datasets, with highly significant results for Duke-Breast-Cancer-MRI (p-value = 0.023) and also CBIS-DDSM (p-value = 1.52E-17). Full ANOVA results are reported in Supplementary Tables S5 and S9 in the Supplementary Information section.

The pre-processing step that differs between the DS and DNS pipelines includes orientation standardization and pixel spacing normalization for the Duke-Breast-Cancer-MRI dataset, as well as maintaining or altering the aspect ratio in square or rectangular formats for the CBIS-DDSM dataset. Through statistical testing, we also observed that these differences were statistically significant. Focusing on each dataset and the best DSC achieved for the CBIS-DDSM dataset, in Fig. 1, we can see some differences between those using Histogram Normalization and Minimum-Maximum, and P1-P99 and Z-Score, with the best score achieved by the DS pipeline using P1-P99 and a 256 $\times$ 256 input size. In the case of the Duke-Breast-Cancer-MRI dataset, the heatmap Fig. 2 shows that the model from the DS pipeline employing Z-Score normalization with a 516 $\times$ 516 $\times$ 64 input size achieved the highest DSC.

We will focus on these two models from each dataset to deeply analyze the impact of the rest of the pre-processing steps.

### Detailed pre-processing methods

Several factors contributed to the model’s performance, but we identified key elements of the pre-processing pipeline that significantly impacted the outcomes. A detailed description of the models and their corresponding pre-processing steps is presented in Supplementary Tables S2 and S7 for each dataset used.

The results suggest that pixel intensity normalization improved segmentation accuracy for both DNS and DS pipelines. When we omitted this step, performance was similar to using Min–Max normalization, likely due to the stabilizing effect of Batch Normalization layers within the network acting on feature maps rather than on per-image Min–Max scaling of the input.

Nevertheless, P1–P99 and Z-Score consistently outperformed both no normalization and Min–Max across image sizes and configurations in the CBIS-DDSM and Duke-Breast-Cancer-MRI datasets. In the CBIS-DDSM dataset, the best performance was achieved in the DS pipeline with P1–P99 normalization and input images resized to 256$\times$256, resulting in a validation DSC of 0.66 and an HD of 27 mm.

For the Duke-Breast-Cancer-MRI dataset, the model that achieved better performance was part of the DS pipeline, precisely the one that used Z-Score as pixel intensity normalization method and that had input images resized to 512 x 512 x 64, resulting in a validation DSC of 0.70 and HD score of 102 mm, as presented in Fig. 2. Focusing on HD, the model from the DS pipeline achieved the best score of 45 mm, using Z-Score as pixel intensity normalization and a 256 x 256 x 64 input size.

Regarding orientation standardization, we assessed its effect as the initial pre-processing step using the CBIS-DDSM dataset, which involved ensuring that all breast images faced the same direction. This analysis indicated that modifying the orientation of mammography images during pre-processing minimally affected the model’s precision in lesion segmentation. A similar evaluation was conducted using the Duke-Breast-Cancer-MRI dataset, where we applied orientation standardization across all images and corresponding segmentations. The results of the impact of the orientation in the DSC and HD for each tissue, namely fat, fibroglandular tissue, and vessels, are shown in the Supplementary Table S3 and S4. It is essential to highlight that small structures, such as vessels or lesions, are more difficult for the model to segment, and small errors produce larger effects on the assessment metrics^31^. Focusing on the model trained with Duke-Breast-Cancer-MRI with the highest DSC, the results indicated that the model using orientation standardization achieved a better validation DSC of 0.71 and an HD of 111 mm, as presented in Table 5. Compared to the same model that did not incorporate this step, we found a statistically significant difference (p-value = 0.0002).

**Table 5:** Validation results for two models from the DS and DNS pipelines, both using input images of size 512$\times$512$\times$64 and Z-Score pixel intensity normalization, with orientation standardization applied in the DS model.

	DSC				HD (in mm)			
Model Name	Total	Breast	FGT	Vessels	Total	Breast	FGT	Vessels
DS 512 Orientation Z-Score	**0.711 ± 0.019**	0.926	0.832	0.374	111 ± 17	150	91	93
DNS 512 Z-Score	0.701 ± 0.018	0.923	0.834	0.346	**106 ± 13**	145	84	90

Terminology	Pipeline	Orientation	Pixel spacing	Pixel intensity	Resize
DS 512 Orientation Z-Score	DS	Yes	No	Z-Score	512 × 512 × 64
DNS 512 Z-Score	DNS	No	No	Z-Score	512 × 512 × 64

The first part of the table reports the mean DSC ± standard deviation and HD ± standard deviation over 5-fold cross-validation on the Duke-Breast-Cancer-MRI dataset, showing both the overall averages (Total) and values for each label (Breast, FGT, Vessels). The second part of the table provides a legend describing the model names.

Bold values denote the best Total results.

As for pixel/voxel spacing normalization, since all images in the CBIS-DDSM dataset had uniform spacing, this aspect was not evaluated for that dataset. However, in the Duke-Breast-Cancer-MRI dataset, which contained varied voxel spacings, we could compare the impact of this pre-processing step on the models. Compared with not doing pixel spacing standardization, the results illustrated that not applying this transformation resulted in a slightly better DSC of 0.70 and worse HD 106 mm, as detailed in Table 6. The evaluation of this impact shows that DSC is significantly different, with a p-value of 0.00014.

**Table 6:** Validation results for two models from the DS and DNS pipelines, both using input images of size 512$\times$512$\times$64 and Z-Score pixel intensity normalization, with pixel spacing normalization applied in the DS model.

	DSC				HD (in mm)			
Model name	Total	Breast	FGT	Vessels	Total	Breast	FGT	Vessels
DS 512 Spacing Z-Score	0.693 ± 0.018	0.919	0.835	0.324	**98 ± 11.3**	127	75	90
DNS 512 Z-Score	**0.701 ± 0.018**	0.923	0.834	0.346	106 ± 13	145	84	90

Terminology	Pipeline	Orientation	Pixel spacing	Pixel intensity	Resize
DS 512 Spacing Z-Score	DS	No	Yes	Z-Score	512 × 512 × 64
DNS 512 Z-Score	DNS	No	No	Z-Score	512 × 512 × 64

The first part of the table reports the mean DSC ± standard deviation and HD ± standard deviation over 5-fold cross-validation on the Duke-Breast-Cancer-MRI dataset, showing both the overall averages (Total) and values for each label (Breast, FGT, Vessels). The second part of the table provides a legend describing the model names.

Bold values denote the best Total results.

Another important step was the padding transformation for the CBIS-DDSM dataset, where mammography images presented rectangular shapes. We tried different strategies to transform these images into square formats and evaluate their effects on model performance, with the results of the different combinations shown in Supplementary Table S8. Our approach involved testing resizing with a maintained aspect ratio versus distorting the images by forcing them into a square shape or adding black pixel padding before resizing. The pre-processing step leading to the best model performance was applying padding, keeping the aspect ratio, and then resizing the images, which achieved a validation DSC of 0.66 and HD of 27 mm, as shown in Table 7. The results evaluating the models’ differences show significant differences with $\alpha$ $<$0.05, with bigger differences between the models DS with size 256 x 256 + intensity normalized with P1-P99 - DNS with size 256 x 448 + intensity normalized with P1-P99 and DNS with size 256 x 256 + intensity normalized with P1-P99 - DNS with size 256 x 448 + intensity normalized with P1-P99. These pairwise differences were determined using T-tests with the Holm-Bonferroni multiple comparisons correction method, and all the results are shown in Supplementary Table S10.

**Table 7:** Validation results for three models from the DS and DNS pipelines on the CBIS-DDSM dataset, using input images of size 256 $\times$ 256 with P1–P99 pixel intensity normalization, while varying resizing strategies (rectangular vs. square shapes, with or without padding).

	Model name	Padding	Keep aspect ratio	DSC ± STD	HD ± STD (in mm)	Comparison		p-value
A	DS 256 P1-P99	Yes	Yes	**0.657 ± 0.269**	**27** ± **14**	A	B	0.004
B	DNS 256 P1-P99	No	No	0.610 ± 0.229	40 ± 21	A	C	0.000
C	DNS 256 448 P1-P99	No	Yes	0.466 ± 0.205	85 ± 19	B	C	0.000

Terminology	Pipeline	Orientation	Pixel spacing	Pixel intensity	Resize
DS 256 P1-P99	DS	Yes	Yes	P1-P99	256 × 256
DNS 256 P1-P99	DNS	Yes	Yes	P1-P99	256 × 256
DNS 256 448 P1-P99	DNS	Yes	Yes	P1-P99	256 × 448

The table reports the mean DSC and HD over 5-fold cross-validation for lesion segmentation, together with their standard deviations. The second part of the table provides a legend describing the model names.

Bold values denote the best results.

Regarding the resizing step, for both datasets, models performed better where input images were resized to smaller dimensions (256 x 256, 512 x 512, and 256 x 256 x 64), as evidenced in Figs. 1 and 2.

Finally, in the pairwise comparisons made using the Holm–Bonferroni method, we observed significant differences when evaluating multiple pre-processing steps at the same time. We then selected the pre-processing steps with the most impact to evaluate their effects more deeply.

For the Duke-Breast-Cancer-MRI dataset, both image resizing and pixel intensity normalization methods significantly affected model performance, with almost all comparisons showing statistically significant differences (p < 0.05), except for one case. The strongest effects were seen between Histogram Normalization and Z-Score (p-value = 7.71E-56) and Histogram Normalization and P1-P99 (p-value = 2.64E-55), highlighting the critical role of normalization strategies, as shown in Supplementary Table S6. For the CBIS-DDSM dataset, image resizing and pixel intensity normalization methods also significantly influenced performance, although several comparisons did not reach significance, underscoring the subtle role of these choices. The most significant effects were observed for image resizing sizes, especially between 1024 vs. 256 (p-value = 2.06E-25) and 1024 vs. 512 (p-value = 4.14E-25), and for pixel intensity normalization methods, particularly Z-Score vs. Minimum-Maximum (p-value = 1.29E-21) and Minimum-Maximum vs. P1-P99 (p-value = 1.50E-19), as shown in Supplementary Table S10.

## Discussion and conclusions

In many studies, there is a lack of detailed information regarding the pre-processing pipelines used and the potential impact these may have on model performance. At the same time, very few studies try to investigate the impact of some transformations on medical imaging, as Shimron et al. did, where they showed that inadequate medical image pre-processing could lead to overly optimistic outcomes^32^, requiring further investigation.

In this study, we investigated the impact of other different pre-processing steps, namely image orientation standardization, pixel/voxel spacing, and intensity normalization (Min-Max, Z-Score, HistNorm, P1-P99), padding, and resizing with different sizes and aspect ratios. We used two different datasets of two breast image modalities (mammography and MRI) to evaluate the influence of such pre-processings on the performance of a simple U-Net model used for lesion and breast tissue segmentation.

One of the pre-processing steps demonstrating a larger influence on model performance was the pixel/voxel intensity normalization. The results demonstrated that while some configurations achieved similar performance metrics, specific pixel/voxel intensity normalization methods positively affected model performance, showing statistically significant differences between them. The models yielding the best performance used P1-P99 or Z-Score, namely the model DS with input size 256 x 256 + intensity normalized with P1-P99 (with CBIS-DDSM dataset achieved 0.66 of DSC) and model DS with input size 512 x 512 x 64 + intensity normalized with Z-Score (with Duke-Breast-Cancer-MRI dataset achieved 0.71 of DSC).

**Fig. 3:**
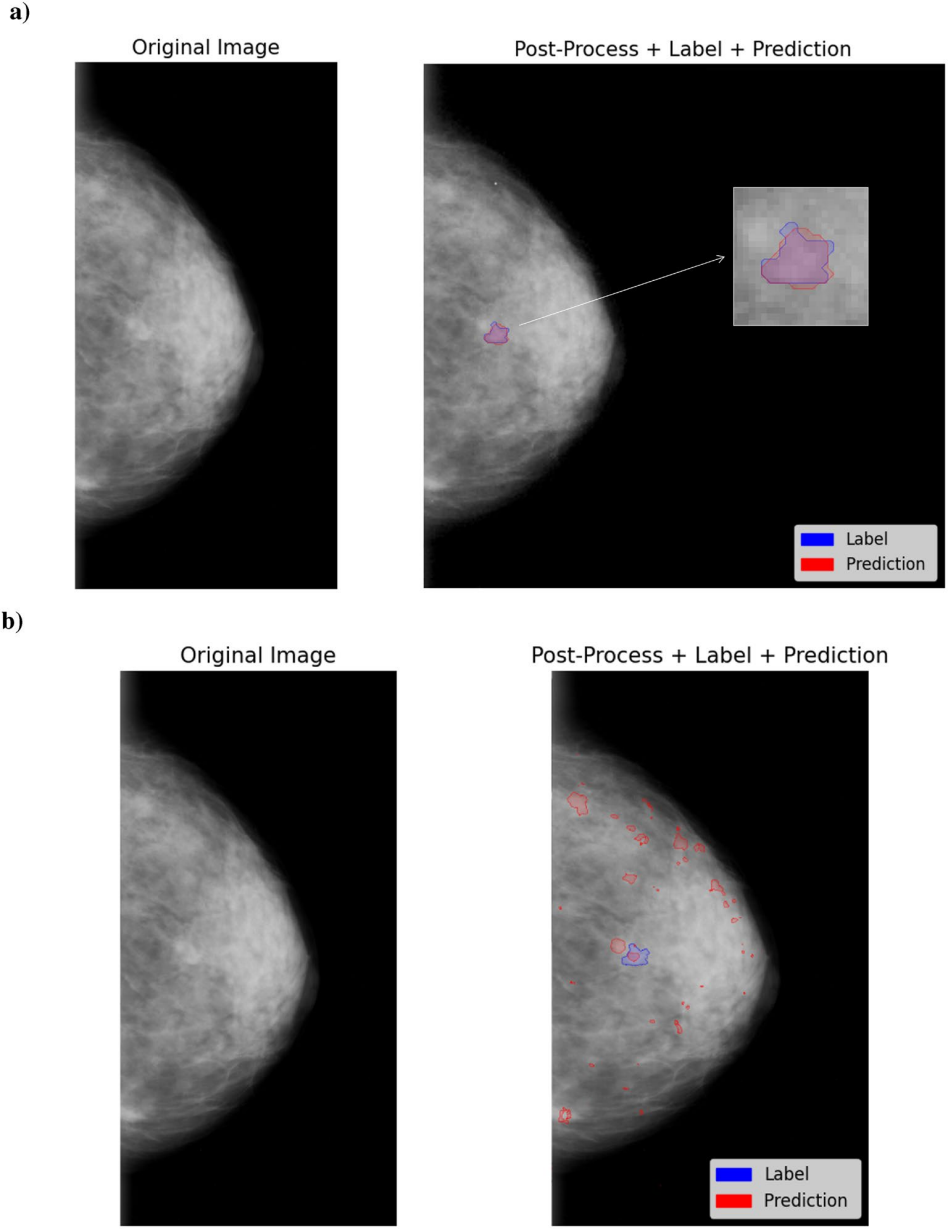
Original image of a patient’s breast from the C6BIS-DDSM dataset, showing: (**a**) post-processed image using the best-performing model, DS with input size 256 x 256 and P1-P99 of intensity normalization, with the label lesion (shown in orange) and the model’s predictions (shown in blue), overlapped on the image; (**b**) post-processed image from the least effective model, DNS with input size 1024 x 1792 and Min-Max of intensity normalization, with the same ground truth label (orange) and the corresponding model predictions (blue), also overlapped.

**Fig. 4:**
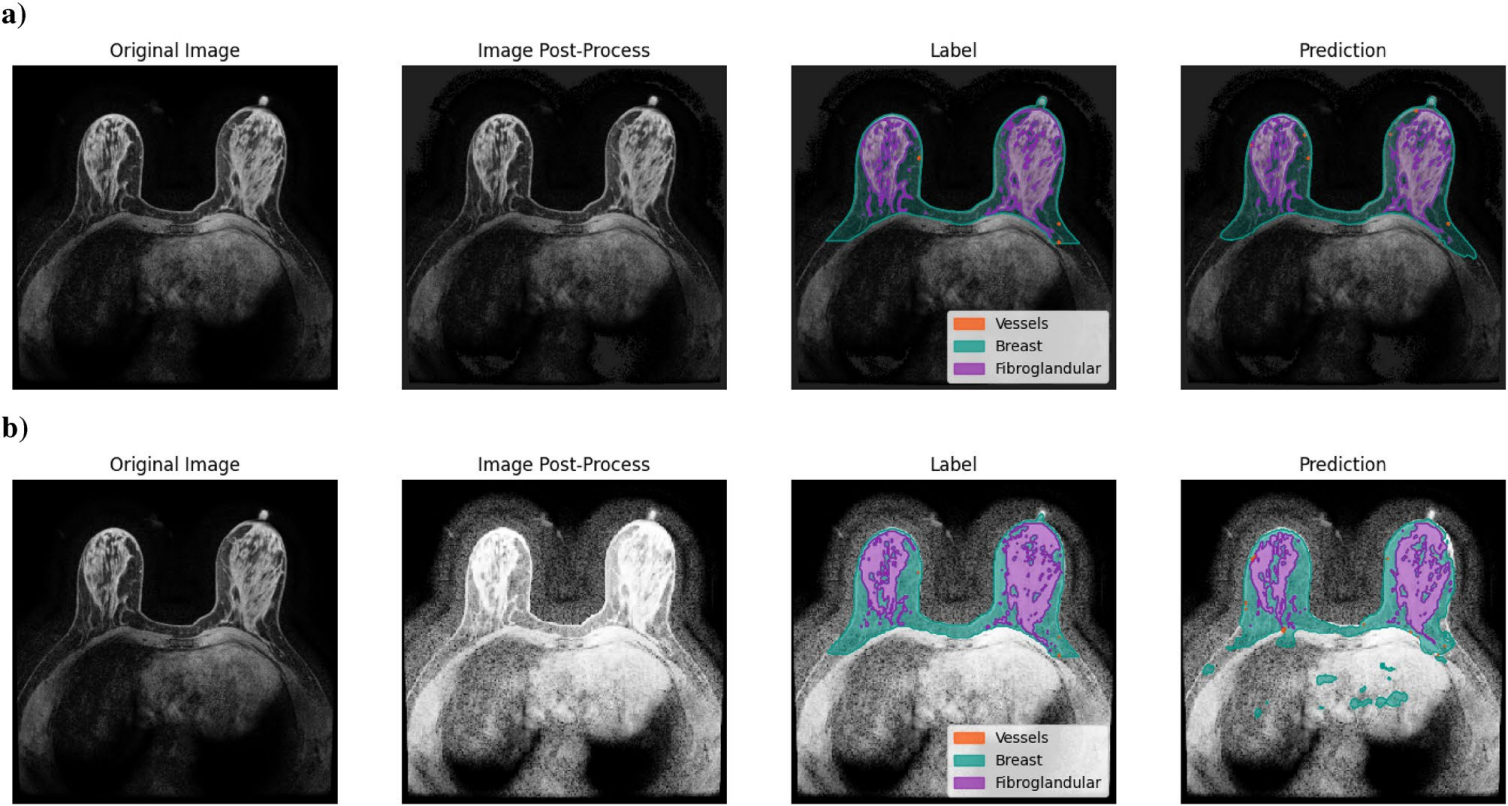
Original image of a patient’s slice from the Duke-Breast-Cancer-MRI dataset, showing: (**a**) post-processed image using the best-performing model, DS with input size 512 x 512 x 64, orientation standardized and Z-Score as intensity normalization, with labels (breast in blue, fibroglandular tissue in yellow, and vessels in orange) and the corresponding model predictions; (**b**) post-processed image using the least effective model, DS with input size of 256 x 256 x 64, spacing standardization and HistNorm of intensity normalization, with the same ground truth annotations and the corresponding model predictions.

As shown in Figs. 3 and 4, we compare the segmentation performance of the best and worst models on representative patient images. For CBIS-DDSM, Fig. 3 displays the original mammogram and the corresponding segmentation results: a) from the best-performing model and b) from the worst-performing one. The best model, DS with input size 256 x 256 and intensity normalized with P1-P99, benefits from a smaller input size and aspect ratio preservation by applying padding to convert the image into a square shape. This pre-processing step, combined with the shallow nature of the model architecture, appears to help retain essential spatial features for accurate lesion segmentation. In contrast, the worst-performing model, DNS with input size 1024 x 1792 and Min-Max as intensity normalization, uses a large input size and struggles to learn meaningful patterns, likely due to its difficulty in handling the scale and complexity of the input. Notably, pixel intensity transformations in both a) and b) do not significantly change visually the image contrast or brightness, suggesting that architectural and input-size choices have a greater impact on segmentation performance than intensity scaling alone. For the Duke-Breast-Cancer-MRI, we present the performance of DS with input size 512 x 512 x 64, orientation standardization, and Z-score as intensity normalization in segmenting the three labels of a specific patient’s slice, compared to the model that showed the lowest DSC. In Fig. 4a, it is evident that both the pre-processed image and the corresponding annotation have good image definition, characterized by strong contrast and well-defined boundaries, which visually makes it easier to differentiate between various tissues. In comparison, Fig. 4b showcases the model with the least performance; here, we can see that the method scales intensities, adding evidence on low intensities (including noise), into the pre-processed image, complicating the model’s ability to distinguish between tissues and negatively affecting prediction accuracy.

Several key factors must be considered when evaluating model performance, including network depth, layer types, and input data quality. In our experiments, we used a relatively shallow U-Net architecture, which likely limited its ability to fully exploit different image resolutions. Nevertheless, we expect that the influence of pre-processing choices would also extend to deeper or more recent models, such as nnU-Net or SwinUNet (a U-Net-like pure Transformer)^33^, as preserving critical image features through appropriate pre-processing remains essential for accurate segmentation.

However, we did not investigate this further as it was outside the scope of our study. We have shared the code for conducting the experiments, which can be adapted for use with more recent architectures or deeper networks.

Although we tested various image sizes (e.g., 256 $\times$ 256, 512 $\times$ 512, 1024 $\times$ 1024), resizing did not consistently produce significant performance changes. This may be due to factors such as the network architecture, task characteristics, target organ or lesion size, and dataset size. For HD, reducing the image size by half resulted in an approximately proportional decrease, reflecting this effect.

Additionally, both datasets used for this study (CBIS-DDSM and Duke-Breast-Cancer-MRI) are relatively small, meaning minor segmentation errors can cause substantial fluctuations in performance. Although the DSC metric is the preferred measure in medical imaging segmentation^34^, it is susceptible when segmenting small structures, where the impact of missing even a single voxel can lead to significant reductions in DSC, as evidenced by the large standard deviations observed in our results. This variability highlights the significant challenges posed by limited data in medical imaging, where even slight changes in model predictions can significantly influence evaluation metrics^31^.

The Duke-Breast-Cancer-MRI dataset model performed better than the CBIS-DDSM models, partly due to the greater difficulty in segmenting small lesions in mammography and differences in image acquisition. Mammography provides non-quantitative information based on X-ray absorption, with pixel intensities reflecting tissue density and radiation dose. Additionally, the high resolution and rectangular shape of CBIS-DDSM images suggest the need for tailored pre-processing.

This study aimed to enhance understanding of how various pre-processing methods and steps might reduce model errors while preserving sensitive information. It is crucial to note that the study’s primary goal was not to achieve the ”best” state-of-the-art model, but to evaluate the effects of each chosen pre-processing step in the DL pipeline for breast image segmentation, specifically using mammography and MRI. By comparing results across different methods, we aimed to clarify how transformations, such as pixel/voxel intensity normalization, image resizing, and others, affected the model’s segmentation performance, suggesting that a more optimal pre-processing pipeline may exist for mammography and MRI.

Also, it is important to evaluate the impact of these methods when deploying these models into clinical practice. These findings show that careful selection and standardization of pre-processing pipelines are crucial, as variations in intensity normalization, image resizing, and orientation standardization can directly affect segmentation accuracy, particularly for small or subtle lesions. For example, inconsistent pre-processing across imaging centers may lead to unpredictable model performance, reducing reliability and limiting generalizability. By fine-tuning the pre-processing techniques for particular modalities, we can achieve higher segmentation accuracy, which promotes a safer and more effective incorporation of AI-assisted segmentation into everyday clinical practices.

Ultimately, this study serves as an initial step by evaluating a limited set of pre-processing methods, while acknowledging that many other approaches, tasks, modalities, and organs remain to be investigated. One limitation of this study is the use of relatively small and specific datasets, which may affect the robustness of the findings. In future work, we aim to expand our analysis to include additional pre-processing techniques, using larger and diverse datasets, and diverse imaging modalities and organs, and to provide a foundation that other researchers can build upon to advance this line of investigation further.

## Supplementary Information

Supplementary Information.

## Data Availability

The datasets analyzed during the current study are available in The Cancer Imaging Archive repository (TCIA). The CBIS-DDSM dataset is available under a CC BY 3.0 license (https://www.cancerimagingarchive.net/collection/cbis-ddsm/), and the Duke-Breast-Cancer-MRI dataset is under a CC BY-NC 4.0 license (https://www.cancerimagingarchive.net/collection/duke-breast-cancer-mri/). Both are publicly accessible and do not require IRB approval for research use.

## Code availability

The code used in this paper can be accessed at https://github.com/jacatarino/breast-imaging-preprocessing.
