# Supplementary material for: The impact of pre-processing techniques on deep learning breast image segmentation
Source: Sci Rep. 2025 Dec 16;16:1095. doi: 10.1038/s41598-025-30724-9 (PMC12789679; doi:10.1038/s41598-025-30724-9)
Supplement: Supplementary file 1 — Supplementary Information. [file 41598_2025_30724_MOESM1_ESM.pdf]

Supplementary Information

Breast_MRI_002	Breast_MRI_008	Breast_MRI_018	Breast_MRI_020	Breast_MRI_047
Breast_MRI_048	Breast_MRI_053	Breast_MRI_057	Breast_MRI_059	Breast_MRI_061
Breast_MRI_064	Breast_MRI_091	Breast_MRI_092	Breast_MRI_102	Breast_MRI_117
Breast_MRI_137	Breast_MRI_145	Breast_MRI_153	Breast_MRI_154	Breast_MRI_162
Breast_MRI_180	Breast_MRI_185	Breast_MRI_196	Breast_MRI_208	Breast_MRI_221
Breast_MRI_222	Breast_MRI_227	Breast_MRI_233	Breast_MRI_243	Breast_MRI_246
Breast_MRI_251	Breast_MRI_255	Breast_MRI_257	Breast_MRI_284	Breast_MRI_286
Breast_MRI_295	Breast_MRI_311	Breast_MRI_316	Breast_MRI_318	Breast_MRI_322
Breast_MRI_329	Breast_MRI_340	Breast_MRI_348	Breast_MRI_351	Breast_MRI_352
Breast_MRI_354	Breast_MRI_355	Breast_MRI_359	Breast_MRI_375	Breast_MRI_383
Breast_MRI_415	Breast_MRI_419	Breast_MRI_423	Breast_MRI_424	Breast_MRI_425
Breast_MRI_434	Breast_MRI_435	Breast_MRI_438	Breast_MRI_444	Breast_MRI_446
Breast_MRI_456	Breast_MRI_462	Breast_MRI_483	Breast_MRI_492	Breast_MRI_516
Breast_MRI_534	Breast_MRI_550	Breast_MRI_552	Breast_MRI_555	Breast_MRI_561
Breast_MRI_565	Breast_MRI_571	Breast_MRI_579	Breast_MRI_590	Breast_MRI_612
Breast_MRI_639	Breast_MRI_657	Breast_MRI_658	Breast_MRI_662	Breast_MRI_664
Breast_MRI_677	Breast_MRI_694	Breast_MRI_709	Breast_MRI_722	Breast_MRI_731
Breast_MRI_741	Breast_MRI_755	Breast_MRI_776	Breast_MRI_780	Breast_MRI_795
Breast_MRI_810	Breast_MRI_821	Breast_MRI_828	Breast_MRI_831	Breast_MRI_861
Breast_MRI_873	Breast_MRI_876	Breast_MRI_886	Breast_MRI_906	Breast_MRI_921

Table S1. List of the 100 patient IDs used from the Duke-Breast-Cancer-MRI dataset.

Pipeline	Orientation Standardization	Pixel Spacing Normalization	Pixel Intensity Normalization	Resize
DNS	No	No	HistNorm	256, 256, 64
DNS	No	No	Min-Max	256, 256, 64
DNS	No	No	P1-P99	256, 256, 64
DNS	No	No	Z-Score	256, 256, 64
DNS	No	No	HistNorm	512, 512, 64
DNS	No	No	Min-Max	512, 512, 64
DNS	No	No	P1-P99	512, 512, 64
DNS	No	No	Z-Score	512, 512, 64
DS	Yes	Yes	HistNorm	256, 256, 64
DS	Yes	Yes	Min-Max	256, 256, 64
DS	Yes	Yes	P1-P99	256, 256, 64
DS	Yes	Yes	Z-Score	256, 256, 64
DS	Yes	No	HistNorm	256, 256, 64
DS	Yes	No	Min-Max	256, 256, 64
DS	Yes	No	P1-P99	256, 256, 64
DS	Yes	No	Z-Score	256, 256, 64
DS	No	Yes	HistNorm	256, 256, 64
DS	No	Yes	Min-Max	256, 256, 64
DS	No	Yes	P1-P99	256, 256, 64
DS	No	Yes	Z-Score	256, 256, 64
DS	Yes	Yes	HistNorm	512, 512, 64
DS	Yes	Yes	Min-Max	512, 512, 64
DS	Yes	Yes	P1-P99	512, 512, 64
DS	Yes	Yes	Z-Score	512, 512, 64
DS	Yes	No	HistNorm	512, 512, 64
DS	Yes	No	Min-Max	512, 512, 64
DS	Yes	No	P1-P99	512, 512, 64
DS	Yes	No	Z-Score	512, 512, 64
DS	No	Yes	HistNorm	512, 512, 64
DS	No	Yes	Min-Max	512, 512, 64
DS	No	Yes	P1-P99	512, 512, 64
DS	No	Yes	Z-Score	512, 512, 64

Table S2. Description of the Duke-Breast-Cancer-MRI models and their associated pre-processing methods. Pixel intensity normalization methods include HistNorm (Histogram Normalization), Min-Max (Minimum–Maximum), and P1-P99 (Percentile 1–Percentile 99). Pipelines are denoted as DS (Domain-Specific, preserving anatomical structure) and DNS (Domain Non-Specific, not preserving anatomical structure).

Pipeline	Resize	Pixel Intensity Normalization	Orientation Standardization	Pixel Spacing Normalization	Total DSC ± STD	Breast DSC ± STD	FGT DSC ± STD	Vessels DSC ± STD
DNS	256, 256, 64	HistNorm	No	No	0.525 ± 0.020	0.778 ± 0.022	0.712 ± 0.031	0.084 ± 0.023
DNS	256, 256, 64	Min-Max	No	No	0.575 ± 0.026	0.815 ± 0.030	0.729 ± 0.060	0.182 ± 0.015
DNS	256, 256, 64	P1-P99	No	No	0.639 ± 0.031	0.917 ± 0.018	0.806 ± 0.035	0.194 ± 0.061
DNS	256, 256, 64	Z-Score	No	No	0.658 ± 0.014	0.917 ± 0.020	0.813 ± 0.024	0.244 ± 0.021
DNS	512, 512, 64	HistNorm	No	No	0.573 ± 0.020	0.817 ± 0.025	0.742 ± 0.024	0.161 ± 0.030
DNS	512, 512, 64	Min-Max	No	No	0.600 ± 0.022	0.851 ± 0.018	0.732 ± 0.035	0.218 ± 0.036
DNS	512, 512, 64	P1-P99	No	No	0.700 ± 0.018	0.917 ± 0.016	0.827 ± 0.025	0.356 ± 0.024
DNS	512, 512, 64	Z-Score	No	No	0.701 ± 0.018	0.923 ± 0.014	0.834 ± 0.025	0.346 ± 0.037
DS	256, 256, 64	HistNorm	Yes	Yes	0.542 ± 0.016	0.820 ± 0.022	0.727 ± 0.023	0.078 ± 0.010
DS	256, 256, 64	Min-Max	Yes	Yes	0.594 ± 0.016	0.860 ± 0.019	0.740 ± 0.041	0.184 ± 0.026
DS	256, 256, 64	P1-P99	Yes	Yes	0.658 ± 0.016	0.923 ± 0.021	0.808 ± 0.026	0.243 ± 0.026
DS	256, 256, 64	Z-Score	Yes	Yes	0.667 ± 0.022	0.918 ± 0.026	0.814 ± 0.034	0.270 ± 0.025
DS	256, 256, 64	HistNorm	Yes	No	0.551 ± 0.016	0.825 ± 0.025	0.734 ± 0.022	0.093 ± 0.006
DS	256, 256, 64	Min-Max	Yes	No	0.591 ± 0.024	0.851 ± 0.022	0.735 ± 0.046	0.185 ± 0.018
DS	256, 256, 64	P1-P99	Yes	No	0.667 ± 0.017	0.923 ± 0.020	0.810 ± 0.031	0.268 ± 0.036
DS	256, 256, 64	Z-Score	Yes	No	0.670 ± 0.020	0.921 ± 0.019	0.819 ± 0.025	0.270 ± 0.027
DS	256, 256, 64	HistNorm	No	Yes	0.522 ± 0.021	0.768 ± 0.022	0.715 ± 0.034	0.083 ± 0.013
DS	256, 256, 64	Min-Max	No	Yes	0.561 ± 0.021	0.813 ± 0.032	0.718 ± 0.036	0.154 ± 0.028
DS	256, 256, 64	P1-P99	No	Yes	0.629 ± 0.021	0.915 ± 0.022	0.808 ± 0.027	0.164 ± 0.033
DS	256, 256, 64	Z-Score	No	Yes	0.652 ± 0.020	0.918 ± 0.019	0.810 ± 0.041	0.226 ± 0.006
DS	512, 512, 64	HistNorm	Yes	Yes	0.582 ± 0.018	0.840 ± 0.027	0.743 ± 0.020	0.164 ± 0.016
DS	512, 512, 64	Min-Max	Yes	Yes	0.602 ± 0.031	0.855 ± 0.027	0.738 ± 0.052	0.213 ± 0.026
DS	512, 512, 64	P1-P99	Yes	Yes	0.697 ± 0.016	0.924 ± 0.011	0.831 ± 0.017	0.334 ± 0.024
DS	512, 512, 64	Z-Score	Yes	Yes	0.705 ± 0.021	0.925 ± 0.019	0.832 ± 0.022	0.356 ± 0.031
DS	512, 512, 64	HistNorm	Yes	No	0.582 ± 0.018	0.840 ± 0.025	0.733 ± 0.032	0.173 ± 0.032
DS	512, 512, 64	Min-Max	Yes	No	0.620 ± 0.023	0.869 ± 0.020	0.750 ± 0.042	0.240 ± 0.022
DS	512, 512, 64	P1-P99	Yes	No	0.708 ± 0.019	0.924 ± 0.015	0.827 ± 0.026	**0.374 ± 0.025**
DS	512, 512, 64	Z-Score	Yes	No	**0.711 ± 0.019**	**0.926 ± 0.018**	0.832 ± 0.027	0.374 ± 0.032
DS	512, 512, 64	HistNorm	No	Yes	0.564 ± 0.020	0.808 ± 0.023	0.734 ± 0.029	0.149 ± 0.027
DS	512, 512, 64	Min-Max	No	Yes	0.593 ± 0.031	0.847 ± 0.019	0.728 ± 0.040	0.204 ± 0.044
DS	512, 512, 64	P1-P99	No	Yes	0.691 ± 0.020	0.915 ± 0.021	0.831 ± 0.018	0.327 ± 0.037
DS	512, 512, 64	Z-Score	No	Yes	0.693 ± 0.018	0.919 ± 0.012	**0.835 ± 0.019**	0.324 ± 0.035

Table S3. Dice Similarity Coefficient (DSC) and Standard Deviation (STD) results for models trained on the Duke-Breast-Cancer-MRI dataset, evaluating all selected pre-processing methods. Pixel intensity normalization methods include HistNorm (Histogram Normalization), Min-Max (Minimum–Maximum), and P1-P99 (Percentile 1–Percentile 99). Pipelines are denoted as DS (Domain-Specific, preserving anatomical structure) and DNS (Domain Non-Specific, not preserving anatomical structure).

Pipeline	Orientation Standardization	Pixel Spacing Normalization	Pixel Intensity Normalization	Resize	Total HD ± STD	Breast HD ± STD	FGT HD ± STD	Vessels HD ± STD
DNS	No	No	HistNorm	256, 256, 64	74 ± 4	96 ± 5	54 ± 4	71 ± 6
DNS	No	No	Min-Max	256, 256, 64	68 ± 7	84 ± 7	55± 10	65 ± 8
DNS	No	No	P1-P99	256, 256, 64	52 ± 8	60 ± 13	45 ± 13	52 ± 9
DNS	No	No	Z-Score	256, 256, 64	50 ± 6	56 ± 14	45 ± 3	**48 ± 5**
DNS	No	No	HistNorm	512, 512, 64	150 ± 17	202 ± 13	94 ± 24	153 ± 32
DNS	No	No	Min-Max	512, 512, 64	124 ± 12	158 ± 10	93 ± 15	121 ± 18
DNS	No	No	P1-P99	512, 512, 64	108 ± 17	145 ± 23	81 ± 23	100 ± 7
DNS	No	No	Z-Score	512, 512, 64	106 ± 13	145 ± 13	84 ± 18	90 ± 19
DS	Yes	Yes	HistNorm	256, 256, 64	76 ± 6	96 ± 9	55± 5	76 ± 7
DS	Yes	Yes	Min-Max	256, 256, 64	65 ± 5	79 ± 6	54 ±10	63 ± 3
DS	Yes	Yes	P1-P99	256, 256, 64	46 ± 7	53 ± 13	36 ± 7	48 ± 8
DS	Yes	Yes	Z-Score	256, 256, 64	**45 ± 10**	**45 ± 13**	40 ± 7	50 ± 10
DS	Yes	No	HistNorm	256, 256, 64	76 ± 7	95 ± 10	51 ± 9	81 ± 9
DS	Yes	No	Min-Max	256, 256, 64	67 ± 11	80 ± 6	55 ± 17	67 ± 12
DS	Yes	No	P1-P99	256, 256, 64	51 ± 8	62 ± 22	**36 ± 2**	53 ± 7
DS	Yes	No	Z-Score	256, 256, 64	50 ± 8	61 ± 20	39± 6	50± 7
DS	No	Yes	HistNorm	256, 256, 64	78 ± 4	96 ± 4	60 ± 3	78 ± 8
DS	No	Yes	Min-Max	256, 256, 64	66 ± 8	83 ± 8	52 ± 10	64 ± 9
DS	No	Yes	P1-P99	256, 256, 64	52 ± 7	64 ± 8	40 ± 7	52 ± 11
DS	No	Yes	Z-Score	256, 256, 64	49 ± 8	51 ± 14	45 ± 6	50 ± 7
DS	Yes	Yes	HistNorm	512, 512, 64	141 ± 9	184 ± 9	87 ± 16	152 ± 17
DS	Yes	Yes	Min-Max	512, 512, 64	128 ± 15	159 ± 6	100 ± 20	126 ± 21
DS	Yes	Yes	P1-P99	512, 512, 64	111 ± 9	150 ± 30	89 ± 6	96 ± 4
DS	Yes	Yes	Z-Score	512, 512, 64	102 ± 23	132 ± 26	84 ± 26	91 ± 21
DS	Yes	No	HistNorm	512, 512, 64	133 ± 8	181 ± 18	76 ± 17	143 ± 12
DS	Yes	No	Min-Max	512, 512, 64	125 ± 8	156 ± 5	92 ± 10	127 ± 13
DS	Yes	No	P1-P99	512, 512, 64	99 ± 22	135 ± 39	70 ± 18	90 ± 24
DS	Yes	No	Z-Score	512, 512, 64	111 ± 17	150 ± 32	91 ± 19	93 ± 15
DS	No	Yes	HistNorm	512, 512, 64	140 ± 13	191 ± 21	91 ± 14	141 ± 21
DS	No	Yes	Min-Max	512, 512, 64	123 ± 7	164 ± 16	87 ± 4	117 ± 5
DS	No	Yes	P1-P99	512, 512, 64	116 ± 16	162 ± 22	86 ± 18	100 ± 20
DS	No	Yes	Z-Score	512, 512, 64	98 ± 11	127± 31	75 ± 14	90 ± 10

Table S4. Hausdorff Distance (HD) and Standard Deviation (STD) results for models trained on the Duke-Breast-Cancer-MRI dataset, evaluating all selected pre-processing methods. Pixel intensity normalization methods include HistNorm (Histogram Normalization), Min-Max (Minimum–Maximum), and P1-P99 (Percentile 1–Percentile 99). Pipelines are denoted as DS (Domain-Specific, preserving anatomical structure) and DNS (Domain Non-Specific, not preserving anatomical structure).

/

Steps	SS	DF	MS	F	p-value uncorrected
orientation	0.227	1.0	0.227	27.544	**0.000**
spacing	0.045	1.0	0.045	5.504	**0.019**
pixel intensity	9.248	3.0	3.083	374.182	**0.000**
resize	1.197	1.0	1.197	145.326	**0.000**
orientation * spacing	0.001	1.0	0.001	0.089	0.765
orientation * pixel intensity	0.006	3.0	0.002	0.23	0.876
spacing * pixel intensity	0.003	3.0	0.001	0.119	0.949
orientation * resize	0.024	1.0	0.024	2.966	0.085
spacing * resize	0.001	1.0	0.001	0.132	0.716
pixel intensity * resize	0.079	3.0	0.026	3.182	**0.023**
orientation * spacing * pixel intensity	0.001	3.0	0.0	0.025	0.995
orientation * spacing * resize	0.001	1.0	0.001	0.133	0.715
orientation * pixel intensity * resize	0.009	3.0	0.003	0.359	0.783
spacing * pixel intensity * resize	0.002	3.0	0.001	0.089	0.966
orientation * spacing * pixel intensity * resize	0.013	3.0	0.004	0.508	0.677

Table S5. 4-factor ANOVA results for Duke-Breast-Cancer-MRI models, assessing the impact of different pre-processing steps. Significant effects ($p < 0.05$) are shown in bold. Table notation: ‘SS’ = sum of squares, ‘DF’ = degrees of freedom, ‘MS’ = mean square, and ‘F’ = F-value.

Steps	A	B	C	p-value uncorrected	p-value corrected
resize	-	256	512	**6.37E-45**	-
pixel intensity	-	HistNorm	Min-Max	-	**3.02E-08**
pixel intensity	-	HistNorm	P1-P99	-	**2.64E-55**
pixel intensity	-	HistNorm	Z-Score	-	**7.71E-56**
pixel intensity	-	Min-Max	P1-P99	-	**8.07E-22**
pixel intensity	-	Min-Max	Z-Score	-	**1.41E-24**
pixel intensity	-	P1-P99	Z-Score	-	**8.35E-09**
resize * pixel intensity	256	HistNorm	Min-Max	-	**9.43E-10**
resize * pixel intensity	256	HistNorm	P1-P99	-	**2.84E-52**
resize * pixel intensity	256	HistNorm	Z-Score	-	**6.40E-54**
resize * pixel intensity	256	Min-Max	P1-P99	-	**2.47E-16**
resize * pixel intensity	256	Min-Max	Z-Score	-	**7.04E-21**
resize * pixel intensity	256	P1-P99	Z-Score	-	**3.66E-13**
resize * pixel intensity	512	HistNorm	Min-Max	-	**2.71E-05**
resize * pixel intensity	512	HistNorm	P1-P99	-	**1.36E-51**
resize * pixel intensity	512	HistNorm	Z-Score	-	**1.34E-51**
resize * pixel intensity	512	Min-Max	P1-P99	-	**3.52E-25**
resize * pixel intensity	512	Min-Max	Z-Score	-	**1.91E-26**
resize * pixel intensity	512	P1-P99	Z-Score	-	5.87E-02

Table S6. Pairwise test results using the Holm–Bonferroni method for Duke-Breast-Cancer-MRI models. Pixel intensity normalization methods include HistNorm (Histogram Normalization), Min-Max (Minimum–Maximum), and P1-P99 (Percentile 1–Percentile 99). Significant differences ($p < 0.05$) are shown in bold.

Pipeline	Orientation Standardization	Pixel Spacing Normalization	Pixel Intensity Normalization	Resize
DS	Yes	Yes	Z-Score	256, 256
DS	Yes	Yes	P1-P99	256, 256
DS	Yes	Yes	Min-Max	256, 256
DS	Yes	Yes	HistNorm	256, 256
DNS	Yes	Yes	Z-Score	256, 256
DNS	Yes	Yes	P1-P99	256, 256
DNS	Yes	Yes	Min-Max	256, 256
DNS	Yes	Yes	HistNorm	256, 256
DNS	Yes	Yes	Z-Score	256, 448
DNS	Yes	Yes	P1-P99	256, 448
DNS	Yes	Yes	Min-Max	256, 448
DNS	Yes	Yes	HistNorm	256, 448
DS	Yes	Yes	Z-Score	512, 512
DS	Yes	Yes	P1-P99	512, 512
DS	Yes	Yes	Min-Max	512, 512
DS	Yes	Yes	HistNorm	512, 512
DNS	Yes	Yes	Z-Score	512, 512
DNS	Yes	Yes	P1-P99	512, 512
DNS	Yes	Yes	Min-Max	512, 512
DNS	Yes	Yes	HistNorm	512, 512
DNS	Yes	Yes	Z-Score	512, 896
DNS	Yes	Yes	P1-P99	512, 896
DNS	Yes	Yes	Min-Max	512, 896
DNS	Yes	Yes	HistNorm	512, 896
DS	Yes	Yes	Z-Score	1024, 1024
DS	Yes	Yes	P1-P99	1024, 1024
DS	Yes	Yes	Min-Max	1024, 1024
DS	Yes	Yes	HistNorm	1024, 1024
DNS	Yes	Yes	Z-Score	1024, 1024
DNS	Yes	Yes	P1-P99	1024, 1024
DNS	Yes	Yes	Min-Max	1024, 1024
DNS	Yes	Yes	HistNorm	1024, 1024
DNS	Yes	Yes	Z-Score	1024, 1792
DNS	Yes	Yes	P1-P99	1024, 1792
DNS	Yes	Yes	Min-Max	1024, 1792
DNS	Yes	Yes	HistNorm	1024, 1792

Table S7. Description of CBIS-DDSM models and their corresponding pre-processing methods. Pixel intensity normalization methods include HistNorm (Histogram Normalization), Min-Max (Minimum–Maximum), and P1-P99 (Percentile 1–Percentile 99). Pipelines are denoted as DS (Domain-Specific, preserving anatomical structure) and DNS (Domain Non-Specific, not preserving anatomical structure).

Pipeline	Orientation Standardization	Pixel Spacing Normalization	Pixel Intensity Normalization	Resize	DSC±STD	HD±STD
DS	Yes	Yes	Z-Score	256, 256	0.564 ± 0.259	31 ± 21
DS	Yes	Yes	P1-P99	256, 256	**0.657 ± 0.269**	**27 ± 14**
DS	Yes	Yes	Min-Max	256, 256	0.494 ± 0.207	86 ± 27
DS	Yes	Yes	HistNorm	256, 256	0.609 ± 0.298	56 ± 18
DNS	Yes	Yes	Z-Score	256, 256	0.601 ± 0.272	38 ± 31
DNS	Yes	Yes	P1-P99	256, 256	0.610 ± 0.229	40 ± 20
DNS	Yes	Yes	Min-Max	256, 256	0.425 ± 0.182	106 ± 38
DNS	Yes	Yes	HistNorm	256, 256	0.345 ± 0.080	100 ± 1
DNS	Yes	Yes	Z-Score	256, 448	0.623 ± 0.213	61 ± 29
DNS	Yes	Yes	P1-P99	256, 448	0.466 ± 0.205	85 ± 19
DNS	Yes	Yes	Min-Max	256, 448	0.413 ± 0.184	173 ± 53
DNS	Yes	Yes	HistNorm	256, 448	0.428 ± 0.163	164 ± 58
DS	Yes	Yes	Z-Score	512, 512	0.439 ± 0.143	77 ± 32
DS	Yes	Yes	P1-P99	512, 512	0.532 ± 0.166	79 ± 32
DS	Yes	Yes	Min-Max	512, 512	0.414 ± 0.108	201 ± 33
DS	Yes	Yes	HistNorm	512, 512	0.483 ± 0.276	200 ± 121
DNS	Yes	Yes	Z-Score	512, 512	0.480 ± 0.167	127 ± 59
DNS	Yes	Yes	P1-P99	512, 512	0.480 ± 0.192	114 ± 35
DNS	Yes	Yes	Min-Max	512, 512	0.413 ± 0.111	250 ± 64
DNS	Yes	Yes	HistNorm	512, 512	0.336 ± 0.085	245 ± 77
DNS	Yes	Yes	Z-Score	512, 896	0.504 ± 0.157	164 ± 42
DNS	Yes	Yes	P1-P99	512, 896	0.405 ± 0.140	221 ± 70
DNS	Yes	Yes	Min-Max	512, 896	0.380 ± 0.148	360 ± 86
DNS	Yes	Yes	HistNorm	512, 896	0.413 ± 0.130	335 ± 56
DS	Yes	Yes	Z-Score	1024, 1024	0.397 ± 0.089	255 ± 26
DS	Yes	Yes	P1-P99	1024, 1024	0.392 ± 0.102	247 ± 66
DS	Yes	Yes	Min-Max	1024, 1024	0.309 ± 0.138	483 ± 131
DS	Yes	Yes	HistNorm	1024, 1024	0.381 ± 0.113	410 ± 33
DNS	Yes	Yes	Z-Score	1024, 1024	0.380 ± 0.107	320 ± 32
DNS	Yes	Yes	P1-P99	1024, 1024	0.374 ± 0.077	288 ± 35
DNS	Yes	Yes	Min-Max	1024, 1024	0.303 ± 0.087	595 ± 61
DNS	Yes	Yes	HistNorm	1024, 1024	0.326 ± 0.064	516 ± 77
DNS	Yes	Yes	Z-Score	1024, 1792	0.361 ± 0.089	537 ± 115
DNS	Yes	Yes	P1-P99	1024, 1792	0.324 ± 0.088	656 ± 121
DNS	Yes	Yes	Min-Max	1024, 1792	0.272 ± 0.074	888 ± 135
DNS	Yes	Yes	HistNorm	1024, 1792	0.327 ± 0.089	943 ± 112

Table S8. Dice Similarity Coefficient (DSC) and Hausdorff Distance (HD) along with respective Standard Deviations (STD) results for models trained on the CBIS-DDSM dataset, evaluating all selected pre-processing methods. Pixel intensity normalization methods include HistNorm (Histogram Normalization), Min-Max (Minimum–Maximum), and P1-P99 (Percentile 1–Percentile 99). Pipelines are denoted as DS (Domain-Specific, preserving anatomical structure) and DNS (Domain Non-Specific, not preserving anatomical structure).

Steps	SS	DF	MS	F	p-value uncorrected
pipeline	16.41228	1	16.41228	174.188	**1.25E-39**
pixel intensity	43.96437	3	14.65479	155.5352	**8.11E-100**
resize	118.3887	2	59.19433	628.2454	**1.72E-266**
pipeline * pixel intensity	16.88282	3	5.627607	59.7273	**1.86E-38**
pipeline * resize	3.331551	2	1.665775	17.67932	**2.13E-08**
pixel intensity * resize	8.632361	6	1.438727	15.2696	**1.52E-17**
pipeline * pixel intensity * resize	7.009418	6	1.168236	12.39881	**5.40E-14**

Table S9. 3-factor ANOVA results for CBIS-DDSM models, evaluating the impact of different pre-processing steps. Significant effects ($p < 0.05$) are shown in bold. Table notation: 'SS' = sum of squares, 'DF' = degrees of freedom, 'MS' = mean square, and 'F' = F-value.

Steps	A	B	C	p-value corrected
resize	-	1024	256	**2.06E-25**
resize	-	1024	512	**4.14E-25**
resize	-	256	512	**3.29E-15**
pixel intensity	-	HistNorm	Min-Max	5.22E-02
pixel intensity	-	HistNorm	P1-P99	**6.96E-08**
pixel intensity	-	HistNorm	Z-Score	**6.55E-12**
pixel intensity	-	Min-Max	P1-P99	**1.50E-19**
pixel intensity	-	Min-Max	Z-Score	**1.29E-21**
pixel intensity	-	P1-P99	Z-Score	5.22E-02
resize * pixel intensity	1024	HistNorm	Min-Max	**2.08E-03**
resize * pixel intensity	1024	HistNorm	P1-P99	9.17E-01
resize * pixel intensity	1024	HistNorm	Z-Score	1.63E-01
resize * pixel intensity	1024	Min-Max	P1-P99	**2.08E-09**
resize * pixel intensity	1024	Min-Max	Z-Score	**8.92E-13**
resize * pixel intensity	1024	P1-P99	Z-Score	3.27E-01
resize * pixel intensity	256	HistNorm	Min-Max	9.17E-01
resize * pixel intensity	256	HistNorm	P1-P99	**4.96E-17**
resize * pixel intensity	256	HistNorm	Z-Score	**2.41E-23**
resize * pixel intensity	256	Min-Max	P1-P99	**5.35E-19**
resize * pixel intensity	256	Min-Max	Z-Score	**2.23E-19**
resize * pixel intensity	256	P1-P99	Z-Score	1.63E-01
resize * pixel intensity	512	HistNorm	Min-Max	9.70E-01
resize * pixel intensity	512	HistNorm	P1-P99	**9.69E-06**
resize * pixel intensity	512	HistNorm	Z-Score	**4.21E-06**
resize * pixel intensity	512	Min-Max	P1-P99	**4.00E-09**
resize * pixel intensity	512	Min-Max	Z-Score	**2.42E-07**
resize * pixel intensity	512	P1-P99	Z-Score	9.70E-01

Table S10. Pairwise test results using the Holm–Bonferroni method for CBIS-DDSM models. Pixel intensity normalization methods include HistNorm (Histogram Normalization), Min-Max (Minimum–Maximum), and P1-P99 (Percentile 1–Percentile 99). Significant differences ($p < 0.05$) are shown in bold.
